# Egg and saturated fat containing breakfasts have no acute effect on acute glycemic control in healthy adults: a randomized partial crossover trial

**DOI:** 10.1038/s41387-021-00176-x

**Published:** 2021-11-09

**Authors:** Chathurika S. Dhanasekara, John A. Dawson, Martin Binks, Allison Childress, Nikhil V. Dhurandhar

**Affiliations:** 1 Department of Nutritional Sciences, Texas Tech University, Lubbock, TX USA; 2 Nutrition and Metabolic Health Initiative, Texas Tech University, Lubbock, TX USA

**Keywords:** Type 2 diabetes, Type 2 diabetes

## Abstract

**Background/Objectives:**

High egg consumption is associated with poor glycemic control. Considering the widespread consumption of eggs, it is crucial to determine causality in this association. We tested if egg consumption acutely alters glucose disposal in the absence or presence of saturated fat, which is frequently consumed with eggs.

**Subjects/Methods:**

In a randomized partial crossover clinical trial, 48 subjects (consuming ≥ 1 egg/week) received two of four isocaloric, macronutrient-matched breakfasts. The groups were defined based on the main ingredient of the breakfasts offered: eggs (EB); saturated fat (SB); eggs and saturated fat (ES); and control, which included a cereal based breakfast (CB). The breakfasts were offered in two testing sessions spaced seven days apart. Six blood samples (pre breakfast (fasting); 30, 60, 90, 120, and 180 minutes post breakfast) were collected to measure glucose and insulin levels. Area under the curves (AUC) were analyzed controlling for the baseline concentrations using mixed-effects models accounting for within-subject dependencies to compare these across breakfast assignments.

**Results:**

Forty-eight patients (46% males, age 25.8 ± 7.7 years, BMI 25.7 ± 4.6 kg/m^2^) were included. Neither EB, SB nor ES was associated with a significant difference in AUC of glucose or insulin compared to CB (*p* > 0.1).

**Conclusions:**

Acutely, consumption of egg breakfast with or without accompanying saturated fat does not adversely affect glucose disposal in healthy adults. While this is reassuring for continued egg consumption, a long-term evaluation of egg intake with or without saturated fat would be the next step.

## Introduction

Eggs are an affordable and nutritionally dense food source. Among the clinical benefits, eggs have a satiating effect, which seems to enhance weight loss [1, 2]. Furthermore, some interventional studies have shown beneficial effects of long-term egg consumption on glycemic control, especially among patients with diabetes, metabolic syndrome or obesity [3–5]. These beneficial effects were evident particularly in the presence of concurrent calorie restriction [3, 4]. In contrast, some recent epidemiological studies have indicated that higher egg consumption may be associated with an increased risk of developing diabetes [6–8] or increased cardiovascular disease (CVD) risk in individuals with diabetes [9, 10]. Therefore, the evidence regarding the effects of egg consumption on glycemic control remains ambiguous.

One plausible explanation for this ambiguity lies in the fact that eggs are often consumed with foods with a high saturated fat content such as bacon or sausage [11]. As such, high egg intake could simply be associated with dietary indiscretions, such as high saturated fat intake, which is positively associated with insulin resistance [11–13]. Therefore, it is possible that the combination of saturated fat and eggs may contribute to worsen glycemic control and negatively impact health [14]. As such, it is unclear if consumption of eggs per se has any detrimental effects on glycemic control.

Establishing the acute effects of egg consumption and associated dietary patterns on glucose disposal is a necessary first step in understanding the long-term effects of eggs on glycemic control. Moreover, understanding the acute effects of eggs and saturated fat, when administered in isolation or in combination, is important for the dietary management of clinical conditions that require tight glycemic control (e.g., gestational diabetes). Therefore, as the next logical and translational step in establishing a causal relationship between egg consumption and glycemic control, we investigated if egg intake per se or the saturated fat that is often consumed with eggs, alter glycemic control in a group of individuals. We hypothesized that the purported association between egg consumption and poor glycemic control is not due to egg consumption per se, but is due to saturated fat consumed along with eggs that affects the glycemic control. To test causality, we determined the effects of eggs and saturated fat on glucose homeostasis in an acute setting by administering four isocaloric macronutrient-matched breakfasts to healthy individuals in a randomized partial crossover clinical trial.

## Materials and methods

### Ethics

The study was approved by the intuitional review board at Texas Tech University (TTU) (IRB2017-215) and was registered with Clinicaltrials.gov (http://www.clinicaltrials.gov/) #NCT 03404700. This study was a part of a larger study that also included an observational component [15]. Power calculation was conducted to the observational part of the study, which was > 80% powered with alpha of 0.05. All the participants were provided written informed consent.

### Subjects

A total of 48 subjects were included in this study following screening for eligibility based on pre-specified inclusion and exclusion criteria. To be included in the study, subjects were required to meet the following criteria: (1) fasting glucose < 126 mg/dl, (2) male or female, (3) BMI 20–60 kg/m^2^, (4) 18–65 years, (5) consume ≥ 1 egg per week for last 3 months. Individuals diagnosed with diabetes or history of gestational diabetes; on antidiabetic medication; pregnant or lactating; history of drug abuse or eating disorders or mental disorder, hypothyroidism or hyperthyroidism, familial hyperlipidemias; allergies or consuming < 1 egg per week; attempting to lose weight; or who were on medications that may influence the study (e.g., antibiotics, anti-depressants, obesity medications) were excluded.

### Study design

The study was conducted as a randomized partial crossover trial that included two study visits. Neither participants nor investigators were blinded to the intervention. Four test breakfasts were matched for total energy and macronutrient composition (Table 1). The groups were divided based on the main ingredient of the breakfasts offered: EB: eggs; SB: saturated fat; ES: eggs and saturated fat; and, CB: control with either 1-eggs (EB), 2-saturated fat (SB), 3-eggs and saturated fat (ES) and, 4-a control (CB).

**Table 1:** Macronutrient composition of test breakfasts.

	Egg breakfast (EB)	Saturated fat breakfast (SB)	Egg and saturated fat breakfast (ES)	Control breakfast (CB)
Energy (kcal)	437.4	452.5	452.3	454.0
Protein (g)	22.5	20.7	22.6	19.9
Fat (g)	23.0	21.7	25.9	19.5
Saturated fat (g)	8.2	11.3	12.9	6.8
Carbohydrates (g)	40.2	46.8	38.7	52.8
Fiber (g)	8.0	6.4	8.0	7.2
GI	53.8	65.1	54.3	65.7
GL	17.3	26.3	16.7	30.0
Weight (g)	313.0	291.5	310.0	304.0
Energy density (kcal/g)	1.4	1.6	1.5	1.5

*GI* Glycemic index, *GL* Glycemic load.

**Fig. 1:**
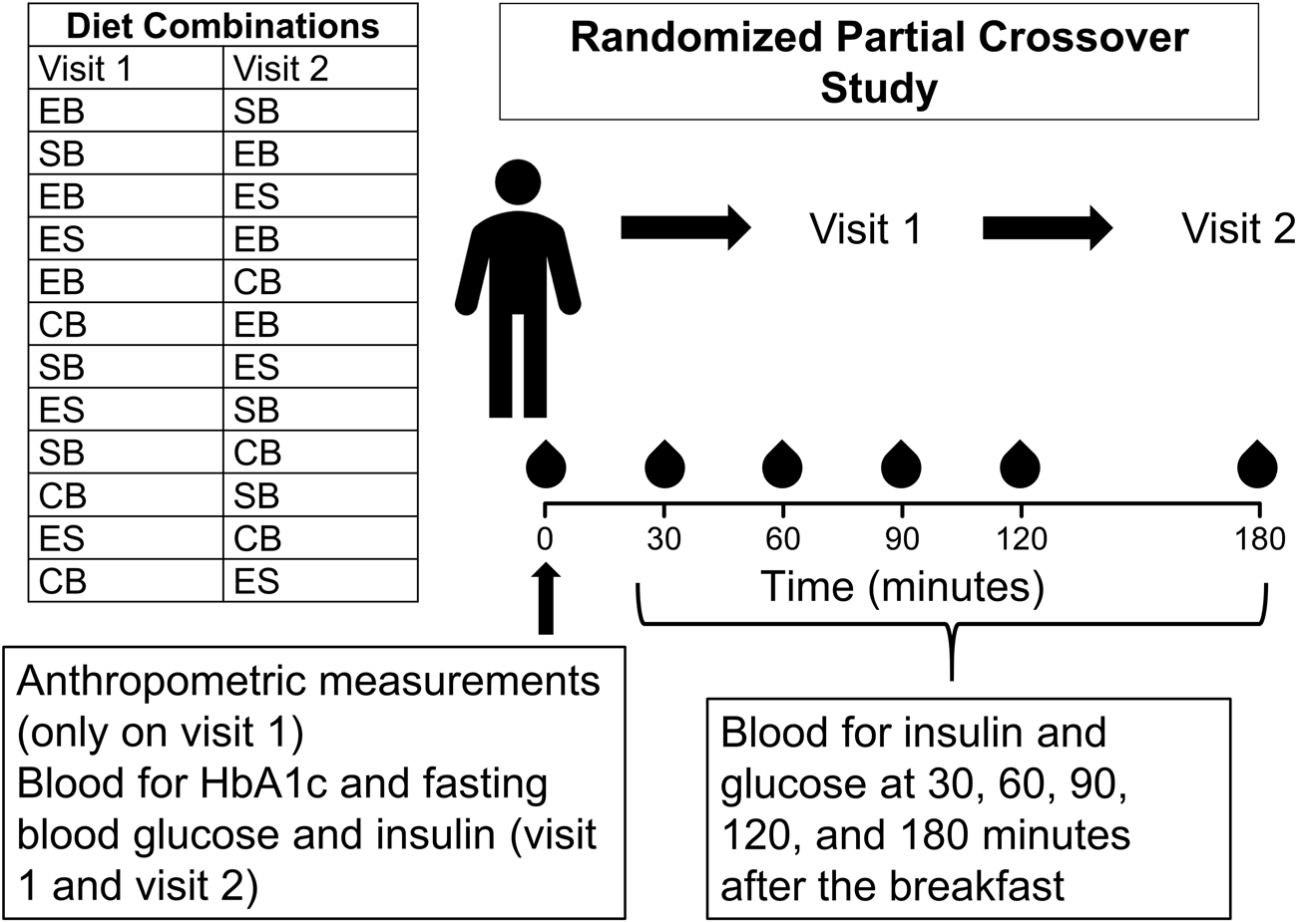
Study flow diagram. EB Egg breakfast, SB Saturated fat breakfast, ES Egg and saturated fat breakfast, CB Control breakfast.

Each subject received only two out of four breakfasts. The 48 subjects were evenly allocated across the 12 possible diet combinations (i.e., EB followed by SB, SB followed by EB, EB followed by ES, ES followed by EB, EB followed by CB, CB followed by EB, SB followed by ES, ES followed by SB, SB followed by CB, CB followed by SB, ES followed by CB, and CB followed by ES) (Fig. 1). Subjects who withdrew or were excluded after starting the study were replaced (same combination assignment) to ensure partial crossover balance.

### Test diets

Four test breakfasts were matched for energy and macronutrient composition (Table 1). Breakfasts were prepared and assembled onsite at the clinical research facility at TTU-NMHI. Preparing and serving meals was done according to a standardized protocol and in compliance with food safety regulations. Weighing of the breakfasts was done using the Mettler-Toledo XS 2002S (Mettler-Toledo, LLC, Columbus, OH). Composition of each breakfast is shown in Table 2. Glycemic index (GI) for each food item was obtained [16] and calculated for each meal [17]. Glycemic load (GL) was calculated using GI of the meal and available carbohydrate content [18, 19].

**Table 2:** Contents of test breakfasts.

Egg breakfast (EB)	Saturated fat breakfast (SB)	Egg and saturated fat breakfast (ES)	Control breakfast (CB)
2 Scrambled Lucerne Fresh Grade AA Large Eggs (Lucerne Foods, Boise, ID, USA)	44 g (1 cup) Kellogg’s Special K Protein, Cereal (Kellogg’s, Battle Creek, MI, USA)	2 Scrambled Lucerne Fresh Grade AA Large Eggs (Lucerne Foods, Boise, ID, USA)	44 g (1 cup) Kellogg’s Special K Protein, Cereal (Kellogg’s, Battle Creek, MI, USA)
120 mL Lucerne Skim Milk (Lucerne Foods, Boise, ID, USA)	200 mL Silk Original Soymilk (White Wave Foods, Denver, CO, USA)	120 mL Lucerne 2% milk (Lucerne Foods, Boise, ID, USA)	200 mL Silk Original Soymilk (White Wave Foods, Denver, CO, USA)
56 g (2 Slices) Nature’s Own Double Fiber Wheat Bread (Flowers Foods Baking Co, Thomasville, GA, USA)	21.5 g (1/2 Slice) Alvarado Street Organic Sprouted Wheat and Oat Bread (Alvarado Street Bakery, Petaluma, CA, USA)	56 g (2 Slices) Nature’s Own Double Fiber Wheat Bread (Flowers Foods Baking Co, Thomasville, GA, USA)	26 g (1 Slice) Mrs. Baird’s Extra Thin Bread (Bimbo Bakeries USA, Horsham, PA, USA)
14 g Signature Kitchens Margarine (Better Living Brands LLC., Pleasanton, CA, USA)	15 g Anchor Salted Pure New Zealand Butter (Fonterra Foodservices (USA) INC., Rosemont, IL, USA)	15 g Anchor Salted Pure New Zealand Butter (Fonterra Foodservices (USA) INC., Rosemont, IL, USA	18 g Signature Kitchens Margarine (Better Living Brands LLC., Pleasanton, CA, USA)
18 g Smucker’s Strawberry Jam (The J.M. Smucker Company, Orrville, OH, USA)		15 g Smucker’s Strawberry Jam (The J.M. Smucker Company, Orrville, OH, USA)	10 g Smucker’s Sugar-Free Strawberry Jam (The J.M. Smucker Company, Orrville, OH, USA)

GI of the meal = Sum of (GI × available carbohydrate for each food)/total available carbohydrate for the meal.

GL = GI of the meal × available carbohydrate of each meal)/100

### Procedure

The study participants presented for visit 1 after a 10-hours fast. After obtaining written informed consent, anthropometric measurements were taken. Height and weight were measured using a wall-mounted Charder HM: 200 P stadiometer (Charder Electronic Co. Ltd. Taichung City, Taiwan) and TANITA MC-780U multi-frequency segmental body composition analyzer (TANITA Corporation of America Inc. Arlington Heights, IL), respectively. FFQ adapted from diet history questionnaire (DHQ) by National Cancer Institute [20], was used to identify subjects belonging to quartiles for egg consumption. The number of eggs eaten per week was determined based on FFQ.

Fasting blood sample was obtained by placing an intravenous catheter. Subjects were provided a test breakfast (from Table 1) and were asked to consume it within 10 min. Blood samples were also obtained at 30, 60, 90, 120, and 180 min after end of consumption of the breakfast. After seven days, participants returned for visit 2 following a 10-hours fast. All procedures performed in visit 2 were similar to visit 1 except for the difference in the test breakfast.

A total of 10 mL of blood was collected at each pre-and postprandial blood draw. Serum was separated by centrifugation at 3500 x *g* for 15 min at 4 °C using Sorvall ST 16 R centrifuge (Thermo Fisher Scientific, Langenselbold, Germany) and stored at −80 °C until analyzed. Aliquots of serum samples were stored at −80 °C until analyzed. For blood glucose levels, a drop of blood was added immediately after collection to blood glucose test strip and read using CONTOUR^®^ Next EZ meter (Ascensia Diabetes Care US, Inc., Parsippany, NJ). Similarly, HbA1c was measured using the A1CNow+ Professional Multi-test HbA1c system (Polymer Technology Systems, Inc., Indianapolis, IN). Samples were handled, stored, and analyzed in accordance with institutional biosafety protocols. Serum insulin levels were measured using a human insulin ELISA kit (Cat#EZHI-14K, EMD Millipore Corporation, Darmstadt, Germany) according to the manufacturer’s protocol. The intra- and inter-assay coefficients of variation for insulin provided by the manufacturer were 6.0%, and 10.3%, respectively. Each sample was run in duplicate.

### Outcomes

Difference of blood glucose levels compared between different test breakfasts. Changes in concentration (area under the curve; AUC) before initiating the breakfast (0 min) to 180 min after consumption of test breakfasts were assessed.

Difference of blood insulin levels compared between different test breakfasts. Changes in concentration (area under the curve; AUC) before initiating the breakfast (0 min) to 180 min after consumption of test breakfasts were assessed.

### Statistical analysis

Data analyses were conducted using R statistical software (version 3.5.3, R Foundation for Statistical Computing, Vienna, Austria) [21]. Descriptive statistics of all outcome variables were computed and tabulated. Missing and biologically implausible values of all measurements were imputed via multiple imputations (20 datasets imputed, 50 iterations per imputation) using the *mice* package (version 3.5.0) in R statistical software [22]. When subsequent analyses involved at least one variable with missing values, analyses were performed on all 20 datasets, and the outcomes were pooled via a random-effects approach. Type I error rate was maintained at 0.05 for each tested hypothesis using the Holm–Bonferroni approach.

Given the partial crossover nature of the study design, carrying the last observation forward (i.e., an intention to treat approach) is likely to bias the group means of only certain participants. As such, to ensure a perfect balance, we decided a priori to replace the defaulting participants with new participants. Furthermore, due to the acute and short-lasting nature of the intervention, attrition is unlikely to be affected by the interventions and as such, could be considered as missing completely at random (MCAR).

Measurements of glucose and insulin during each visit were used to determine area under the curves (AUC) of glycemic control. AUC of glycemic control were regressed on the presence of eggs, saturated fat and their interaction in the test breakfast controlling for the measure of interest at baseline in a mixed-effects model constructed using the *lme4* package (version 1.1-21) [23] and *lmerTest* package (version 3.1-0) [24] in R statistical software. Code is available as a **Supplement**.

## Results

**Fig. 2:**
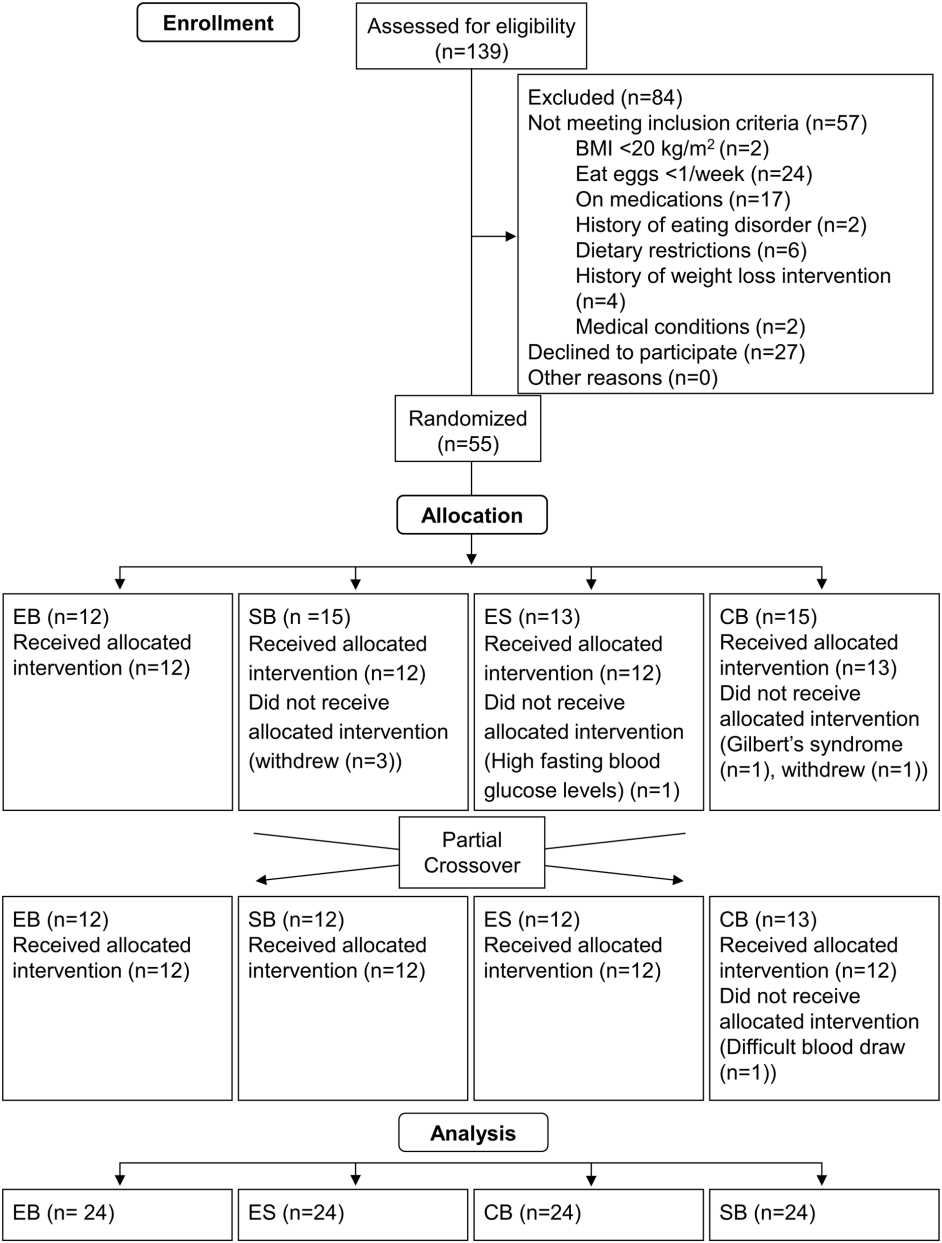
CONSORT diagram. EB Egg breakfast, SB Saturated fat breakfast, ES Egg and saturated fat breakfast, CB Control breakfast.

One-hundred and thirty-nine subjects were screened for eligibility. Eighty-four subjects were excluded for not meeting eligibility criteria or for declining to participate. Another seven participants were excluded during the study. The details of study recruitment are shown in the CONSORT diagram (Fig. 2). Descriptive characteristics of the study sample and each test group are presented in Table 3. Mean age of the whole study sample was 25.8 ± 7.7 years and 46% were males. The age of subjects ranged from 19 to 58 years. There were, 52.1% Caucasian, 31.3% Asian, and 16.7% Hispanic. The mean BMI of the whole sample was 25.7 ± 4.6 kg/m^2^ with a range of 20.1–38.0 kg/m^2^.

**Table 3:** Sample characteristics in the whole sample.

	Egg breakfast (EB) *n* = 24	Saturated fat breakfast (SB) *n* = 24	Egg and saturated fat breakfast (ES) *n* = 24	Control breakfast (CB) *n* = 24	Whole sample *n* = 48
Age (years)	26.4 ± 7.5	27.2 ± 10.1	23.8 ± 3.8	26.0 ± 7.7	25.8 ± 7.7
Sex (males)	14 (58.3%)	13 (54.2%)	7 (29.2%)	10 (41.2%)	22 (45.8%)
Weight (kg)	76.9 ± 16.2	79.5 ± 21.6	72.0 ± 20.8	77.8 ± 16.9	76.6 ± 19.0
Height (m)	172.3 ± 10.9	173.6 ± 10.5	167.9 ± 10.4	171.6 ± 10.7	171.4 ± 10.8
BMI (kg/m^2^)	25.7 ± 4.0	26.0 ± 5.3	25.0 ± 4.9	26.2 ± 4.3	25.7 ± 4.6
Fasting blood glucose (mg/dL)	95.6 ± 9.8	97.3 ± 7.5	93.4 ± 8.4	96.9 ± 6.9	95.8 ± 9.0
Fasting insulin level (µIU/dL)	7.2 ± 4.64	8.6 ± 7.8	9.4 ± 9.6	8.7 ± 10.2	8.5 ± 8.8
HbA1c (%)	5.3 ± 0.5	5.4 ± 0.3	5.3 ± 0.4	5.2 ± 0.4	5.3 ± 0.4
Routine egg consumption*					
2.9/week	6 (25%)	11 (45.8%)	11 (45.8%)	12 (50%)	20 (41.7%)
3–4.9/week	8 (33.3%)	9 (37.5%)	6 (25%)	5 (20.8%)	14 (29.2%)
5–6.9/week	6 (25%)	3 (12.5%)	5 (20.8%)	5 (20.8%)	10 (20.8%)
≥ 1/week	4 (16.7%)	1 (4.2%)	2 (8.3%)	1 (4.2%)	4 (8.3%)

BMI Body mass index, *Baseline egg consumption according to food frequency questionnaire.

**Fig. 3:**
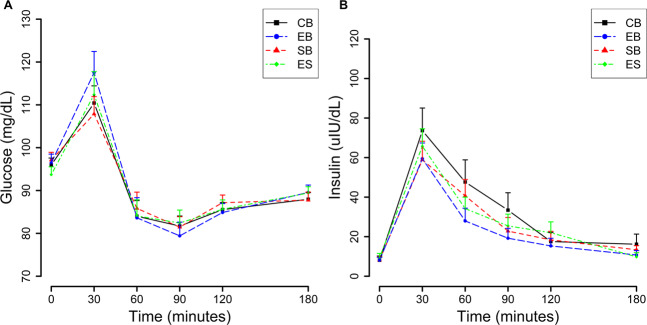
Glucose and insulin responses to different breakfasts (*n* = 24). **A** Glucose in response to breakfasts reported as mean ± SE mg/dL values. **B** Insulin in response to breakfasts reported as mean ± SE µIU/dL values. There were no statistically significant differences between the mean area under the curves (AUC) of glucose or insulin following EB, SB, and ES when compared with CB (*p* > 0.05). EB Egg breakfast, SB Saturated fat breakfast, ES Egg and saturated fat breakfast, CB Control breakfast.

When AUC of glucose was regressed on the administered breakfast group, controlling for the blood glucose levels at the baseline in a mixed-effects model, including the CB as the reference category, there were no statistically significant differences between the mean AUC of glucose following EB, SB, and ES when compared with CB (Cohens *d* = 0.072, 0.06, and 0.200, respectively, *p* > 0.05) (Fig. 3 and Table 4). Similarly, when AUC of insulin was regressed on the administered breakfast controlling for the serum insulin levels at the baseline in a mixed-effects model, including the CB as the reference category, none of the test breakfast vs. CB comparisons of mean AUC of insulin were significant (Cohens *d* = 0.108, 0.130, and 0.113, respectively, *p* ≥ 0.05) (Fig. 3 and Table 4).

**Table 4:** Summary of regression analyses.

Summary of regressing area under the curves (AUC) of glucose (min*mg/dL) values vs. administered breakfast while controlling for blood glucose levels at baseline					
IVs	*β*	SE	df	*t*-statistic	*p*-value
Intercept (CB)	2439.691	1543.242	87.056	1.581	0.118
EB	104.930	305.829	87.056	0.343	0.732
SB	−94.488	305.185	87.056	−0.310	0.758
ES	414.633	433.224	87.056	0.957	0.341
Fasting Glucose Value	143.377	16.026	87.056	8.946	<0.001
Summary of regressing AUC of serum insulin values (min*µIU/dL) vs. administered breakfast while controlling for serum insulin levels at baseline.					
Intercept (CB)	3533.248	779.929	50.209	4.530	<0.001
EB	−397.341	766.053	51.431	−0.519	0.606
SB	−461.760	740.428	57.594	−0.624	0.535
ES	594.812	1102.153	47.817	0.540	0.592
Fasting Insulin Value	223.132	67.286	12.931	3.316	0.006

EB Egg breakfast, SB Saturated fat breakfast, ES Egg and saturated fat breakfast, CB Control breakfast, SE Standard Error, df degrees of freedom.

## Discussion

Despite multiple interventional trials having been conducted to date to examine the effects of egg consumption on glycemic control, the interactive effects of the commonly consumed combination of eggs and saturated fat have not been studied. This study examined the acute effects of eggs, saturated fat, and their combination on glycemic control in a randomized controlled trial after administering isocaloric, macronutrient-matched test breakfasts. Here, we did not observe a significant acute effect of eggs or accompanying saturated fat intake on the acute glycemic response when total energy and macronutrient composition were matched.

These results are contrary to some previous acute interventional studies [25, 26]. It is important to note that most of the previous studies provided total energy matched but not macronutrient matched breakfasts. For instance, Ratliff et al. [25]. showed that an egg breakfast decreases glucose AUC compared to a bagel breakfast where the total energy content was matched but not the macronutrient composition in a group of healthy individuals. The high carbohydrate load in the bagel breakfast (72% of total energy) compared to the egg breakfast (22% of total energy) may have given rise to the difference in glycemic response.

Similarly, Rains et al. [26] showed a difference in acute glycemic response when high protein sausage-based (carbohydrate 4%, protein 53% of total energy) and sausage and egg-based breakfasts (carbohydrate 19%, protein 43% of total energy) were compared with pancake-based breakfast (carbohydrate 61%, protein 4% of total energy). Interestingly, there was no difference in glycemic control between the two high-protein diets when the difference in protein and carbohydrate content were only 9 g and 10 g, respectively. This indicates that when the macronutrient composition become comparable the difference in glycemic response becomes minimal. The breakfast meals provided in our study had around 41% energy from carbohydrate, 18% energy from protein, and 43% energy from fat. Hence, our findings suggest that when controlled for total energy and macronutrient composition, consumption of eggs or saturated fat may not lead to acute impairment of glycemic control. Furthermore, some evidence suggests that routine egg consumption may improve glycemic control [4–6]. Notably, in our study, these subjects are habitual egg consumers thus eliminating the issue of altered glycemic response due to routine egg consumption playing a role in our findings.

Several studies have shown the long-term benefits of egg consumption for glycemic control [4, 5, 27, 28]. All these studies were conducted at least for 12 weeks duration. Therefore, we do not attempt to compare our results (i.e., acute effects of consumption of an egg breakfast) with the potential long-term benefits of egg consumption as suggested in these studies; it is important to note that majority of these studies used carbohydrate-restricted diets for the egg condition. In our study, subjects were not on any dietary restrictions and approximately 41% of the total energy of all breakfasts was from carbohydrates. Our results suggest that at least in an acute setting, the beneficial [3, 5] or often implied detrimental effects [6] of egg consumption on glycemic control are less likely to be apparent after the macronutrient composition of the test and control interventions are matched. Long-term effects of iso-caloric, macronutrient composition, and GI-matched studies need to be conducted to establish/dispute the associations between egg consumption and glycemic control suggested in the aforementioned interventional trials.

Saturated fat consumption is often thought to be associated with poor glycemic control and insulin resistance, especially when combined with eggs [13, 29]. For instance, Koska et al. [29] showed that short-term feeding of a saturated fat-rich diet induces insulin resistance. Similarly, another study showed evidence of developing insulin resistance following a high-fat diet composed of 25% energy from saturated fat (55% energy from fat and 27% energy from carbohydrate) for three weeks compared to a low-fat diet comprised of 8% energy from saturated fat (20% energy from fat and 62% energy from carbohydrate) [30]. Even though saturated fat accounted for approximately 24% of energy in our saturated fat breakfasts, we did not observe significant differences in glucose clearance between saturated fat-containing breakfasts even after combining with eggs. However, it should be noted that the main saturated fat source used in our breakfasts was butter. Recent evidence suggests that dairy fat may be beneficial for glucose homeostasis [31]. For instance, a recent meta-analysis of observational studies suggested that increased consumption of butter appears to be associated with a decreased incidence of diabetes (RR = 0.96 [0.93, 0.99]) [31]. It is unknown if replacing butter in our breakfasts with an alternative source of saturated fat may alter our findings.

The diets were designed by a registered dietitian to resemble typical breakfast meals, and inordinate manipulations of ingredients were avoided. This allowed us to better represent a more generalizable meal scenario while maintaining reasonable control over the most salient dietary factors related to glycemic control. In short, controlling for every single potential variable was impossible as realistic breakfasts were used. Although the four breakfasts were closely matched for energy and macronutrient composition, they were not matched for exact fiber content, glycemic load, or energy density. However, we tried to keep all the parameters including fiber content, glycemic index, and load and energy density as close as possible. When we tried match everything it created a discrepancy in fiber (~2 g of fiber), which is unlikely to cause serious alteration of glycemic response [32]. Similarly, using manufactured food might have influenced the outcome of the study as we cannot control the influence of miscellaneous contents such as presence of artificial sweeteners, soy products, and different types of dietary fibers. However, there is evidence that suggests that differences in dietary fiber content in these test breakfasts had minimal effect on acute glycemic control [32]. Moreover, the glycemic index and glycemic load differences between individual foods become insignificant when mixed meals are consumed [33]. However, this may have an indirect impact on the results as we were assessing acute glycemic control.

Our study had several notable strengths. First, we used iso-caloric breakfasts that were also matched based on the macro-nutrient content. Comparison of macro-nutrient composition-matched diets increases the scientific rigor and hence the validity of our results. Second, implementing a repeated measures crossover design at least partially controlled for within-subject variability of outcome measures and improved the statistical power of the design. Third, measurement of glycemic control via multiple parameters (i.e., insulin and glucose AUC) provided converging evidence regarding the association between egg and saturated fat intake and glycemic control. Fourth, we employed multiple imputations to impute missing data, taking the non-random nature of missingness into consideration. While this approach was time-consuming, despite the high computational power of the computers used for the analyses, the outcomes of the analyses are likely to be less biased. Finally, we used a series of mixed-effects models that accounted for the nested nature of repeated measurements within subjects, while simultaneously comparing all three test breakfasts with CB. This approach improved the generalizability of our findings and minimized type I error rate inflation due to eliminating the need to perform multiple post-hoc comparisons.

Though many studies that examined the potential effects of egg consumption glycemic control were conducted as long-term studies [4, 5, 27, 28] or among individuals with impaired glucose conditions such as diabetes or obesity [25, 26], we conducted our study in healthy individuals in order to capture the actual physiological response in tightly controlled conditions. However, capturing the actual alteration was challenging provided that the effect size would have been smaller than we have anticipated. Furthermore, this study was a part of another study and the power calculation was conducted for the observational part of the study [15]. And based on the effect sizes we observed our study was only 5.7%, 5.5%, and 10.4% powered to statistically significant of difference in acute glycemic response between (1) EB and CB, (2) SB, and CB (3) ES and CB, respectively.

Moreover, having a sample that mainly comprised of college students limited our ability to generalize the null associations. Furthermore, physical activity was not given a consideration for recruitment or as an outcome. Although each subject largely served as their own control (given the crossover design), habitual physical activity would likely influence acute glucose disposal of a meal. Medications such as statins and antihypertensives were not considered when selecting subjects that may influence on glucose metabolism. Moreover, we did not consider whether they were on hormone therapy during or prior to the study. Employing a partial crossover design rather than a full crossover design (due to limitations in funding and to minimize participant burden) limited the statistical power of the design. We are also aware that the lack of acute effect of these breakfasts on glycemic control may not reflect their effect upon long-term consumption. Perhaps, the effect of eggs with or without saturated fat takes time to impact glycemic control. It is also possible that the subjects had glycemic control within normal limits, which allowed them to handle the variations in various test breakfasts easily. Since we measured glucose 30 min apart, this could lead to missing the actual glucose peak at the early postprandial period. However, our glucose and insulin curves showed a possible peak around 30 min.

In conclusion, in healthy adults, breakfasts matched for energy density and nutrient composition, but containing eggs, saturated fat, or the combination of eggs and saturated fat do not affect glucose disposal in an acute setting compared to a control breakfast of similar energy and macronutrient content. Hence, our hypothesis (i.e., the purported association between egg consumption and poor glycemic control is not due to egg consumption per se but is due to saturated fat consumption along with eggs alter glycemic control) was disproved. Since observational studies raised doubt about the possible role of eggs in diabetes, clarity was needed to determine if eggs or eggs with accompanying saturated fat intake would influence glucose disposal. The null association observed in the current study in relation to egg and saturated fat consumption, and glucose disposal shows no particular caution against egg consumption at this time. These findings should inform longer term evaluation of egg consumption on glycemic control.

## Supplementary information

R code
